# The Fight Against Cervical Cancer

**DOI:** 10.1016/j.ebiom.2015.01.001

**Published:** 2015-01-09

Globally, cervical cancer is one of the most deadly forms of cancer affecting women—killing more than 270,000 people every year. The majority of these deaths (85%, according to the WHO) occur in developing countries. A global health priority, therefore, is to provide low-cost, highly effective cervical cancer prevention, detection, and control options to all women who need them.

The human papillomavirus (HPV) family is a group of about 150 related viruses, a subset of which is thought to be responsible for the majority of cervical cancer cases. One of the most successful translational achievements in cervical cancer research in recent years is the development of highly effective preventative vaccines which directly target these oncogenic HPV strains. The bivalent vaccine Cervarix(r) (specific for HPV-16 and -18 strains) and the quadrivalent vaccine Gardasil(r) (which is additionally specific for HPV-6 and -11) contain viral L1 proteins from these strains, which are able to self-assemble into virus-like particles (VLPs). Recently, Gardasil(r) 9 has been approved by the United States FDA which will contain an additional 5 strains of HPV not currently covered by available vaccines. These vaccines induce antibody responses against HPV, but do not contain any of the viral genomic DNA present in natural viruses and, therefore, are not able to establish an infection. Available data indicate that these vaccines are highly effective at preventing precancerous cell induction in women, and since their introduction, global HPV infection rates appear to be declining.

Although these prophylactic vaccines are highly effective at preventing new infections, neither vaccine is capable of inducing immune responses against established HPV infections. A major healthcare goal, currently, is to develop therapeutic approaches to resolving potentially cancerous HPV lesions (otherwise known as cervical intraepithelial neoplasia (CIN), or cervical dysplasia). Several approaches are underway, including a therapeutic vaccine which is likely to work by inducing an adaptive T cell-mediated response against the viral E6 and E7 proteins, which are expressed in these lesions. Several drugs are also being explored, including an antibody (bevacizumab) against vascular endothelial growth factor (VEGF), which may help patients by limiting the growth of new blood vessels to the cancer. Clinical trials are underway to test whether chemotherapy drugs or immune checkpoint inhibitors such as ipilimumab may work well in combination with radiation therapy. Autologous T cell transfers, where the patients own immune cells are “trained” outside of the body to attack cancerous cells infected with HPV are also being explored. Armed with a basic scientific understanding of how HPV replicates and may contribute to cervical cancer, and how the patient's own immune system may be “kicked into gear” in order to attack those cancer cells has given researchers several potential avenues which may already be explored as treatment options.

In addition to established principles about viral replication and immune system function, scientists are also using systems approaches to identify new genetic markers which are common in transformed cervical lesions, and ongoing studies are looking at the basic molecular mechanisms of how HPV-infected cervical cells become cancerous. These basic approaches may help researchers identify and manipulate new molecular pathways that are biologically relevant for cervical cancer transformation, and which may be amenable to therapeutic manipulation.

Although several promising treatment and prevention options are available or under development, early detection remains one of our most effective tools for combating cervical cancer. The Pap test, introduced in the 1950s, is a simple means of screening cervical cells for abnormalities and may now be combined with HPV DNA screening to identify those patients at greatest risk for developing cervical cancer. Although highly effective, the Pap test may not be a practical screening tool for low-resource countries, and there is an urgent need for development and effective implementation of inexpensive, easily-administered screening options for these women—such as HPV DNA screening kits that may not necessarily need expensive refrigeration or a well-equipped laboratory to administer. Visual inspection with acetic acid (VIA) is an inexpensive option currently in practice, but this method may not be as accurate as DNA testing.

We have come a long way since the introduction of the Pap test. Identification of HPV as the major etiological agent of cervical cancer, for example, has allowed rational vaccine design and the development of targeted immunotherapies. However, there is still much work to do to improve practical screening options in developing countries, and to add to our arsenal of treatment options for established HPV infections.

EBioMedicine

